# Enhancing Sustainable Machining of Inconel 718 via Synergistic Coupling of Rehbinder Effect and Heat Transfer Using Active Thermal Conductive Medium

**DOI:** 10.3390/ma19142960

**Published:** 2026-07-09

**Authors:** Qingan Yin, Wangbo Gong, Rui Yang, Siyu Liu, Jinxiao Xu, Jianxiong Chen

**Affiliations:** School of Mechanical Engineering and Automation, Fuzhou University, Fuzhou 350108, China; 2500227159@fzu.edu.cn (W.G.); 2500227118@fzu.edu.cn (R.Y.); 240227122@fzu.edu.cn (S.L.); 240220024@fzu.edu.cn (J.X.); jxchen045@fzu.edu.cn (J.C.)

**Keywords:** Inconel 718, active thermal conductive medium, rehbinder effect, enhance heat conduction

## Abstract

Inconel 718 exhibits poor machinability due to its high strength and low thermal conductivity, which induce severe thermo-mechanical loads. Conventional cooling strategies struggle to concurrently regulate heat dissipation and interface lubrication. This paper proposes a machining method based on Active Thermal Conductive Media (ATCM), which simultaneously exerts the Rehbinder mechanochemical effect and solid-phase enhanced heat transfer effect by pre-coating a liquid graphene film on the workpiece surface. Orthogonal turning tests were conducted using a K313 carbide tool at a cutting speed of 30 m/min, cutting width of 2 mm, and undeformed chip thickness of 0.1 mm. The cutting force, cutting temperature, cutting power, and tool wear characteristics under six machining conditions—dry cutting, flood cutting, Minimum Quantity Lubrication (MQL), Cryogenic MQL (CMQL), Nanofluid MQL (NMQL), and ATCM-assisted cutting—are systematically compared. The results show that ATCM achieves a 21.6% reduction in cutting force, a 20% reduction in cutting temperature, and a 34.9% reduction in cutting power through the synergistic coupling effect of reduced heat generation and enhanced heat dissipation, with adhesive wear and diffusion wear of the cutting tool significantly suppressed.

## 1. Introduction

Hot-end components of aero-engines operate long-term in extreme environments characterized by high temperatures, high pressures, heavy loads, and frequent thermal cycling, imposing stringent requirements on the high-temperature mechanical properties and surface integrity of structural materials [1]. Inconel 718 nickel-based superalloy, owing to its exceptional fatigue resistance, impact toughness, and high-temperature stability, is extensively utilized in core components such as aero-engine turbine disks and combustor blades [2]. However, Inconel 718 exhibits extremely poor machinability due to its low room-temperature thermal conductivity (only ~11.4 W/m·K), high elevated-temperature strength, and strong work-hardening tendency [3]. During cutting, approximately 90% of the plastic deformation energy and frictional energy is converted into heat, which cannot be rapidly dissipated through the workpiece matrix, resulting in extremely localized high temperatures in the cutting zone. This in turn induces severe tool wear, microcracks on the machined surface, and residual stress concentration. Therefore, mitigating the thermo-mechanical loads during the cutting of Inconel 718 is critical for improving its machinability.

To improve the machinability of the difficult-to-cut Inconel 718, researchers have conducted extensive research on cooling and lubrication technologies [4]. Conventional flood cooling achieves heat dissipation through forced convection of a large volume of cutting fluid, but its heat transfer efficiency is severely limited by the Leidenfrost effect [5]. A continuous vapor film formed at the high-temperature interface blocks direct contact between the liquid-phase cutting fluid and the heat source, and the extensive use of cutting fluid also causes environmental pollution and health hazards [6]. Minimum Quantity Lubrication (MQL) technology realizes lubrication and cooling by atomizing a minimal amount of cutting fluid, significantly reducing cutting fluid consumption [7]. However, it is limited by the insufficient heat capacity of the working medium and cannot meet the high heat flux dissipation requirements of Inconel 718 cutting [8]. Cryogenic Minimum Quantity Lubrication (CMQL) and Nanofluid Minimum Quantity Lubrication (NMQL), developed on the basis of MQL, have enhanced cooling and lubrication effects by introducing cryogenic media and high-thermal-conductivity nanoparticles, respectively [9].

For CMQL, Ostrowicki et al. [10] and Bagherzadeh et al. [11] confirmed that CMQL outperformed pure CO_2_ cooling in Inconel 718 machining, achieving 30% longer tool life, 18% lower cutting temperature, and surface roughness below 0.2 μm, verifying its potential as a sustainable alternative to flood cooling. For NMQL, Pan et al. [12] and Namlu et al. [13] demonstrated that nanofluids containing C60 or MWCNT/TiO_2_ nanoparticles significantly reduced specific cutting energy, cutting force and surface roughness via the micro ball-bearing effect and synergistic lubrication-heat transfer mechanisms. Nevertheless, both CMQL and NMQL, as convective cooling technologies, have not broken through the inherent bottlenecks of convective heat transfer.

In recent years, machining technology based on the Rehbinder effect using surface-active media has attracted widespread attention [14]. This technology involves coating a small amount of surface-active media on the workpiece surface, which reduces the material’s surface free energy through chemisorption, enhances dislocation mobility, and promotes ductile-to-brittle transition of the material, thereby significantly reducing cutting forces and surface roughness [15].

Studies using high-speed in situ imaging systems have revealed that the Rehbinder effect induces changes in the plastic flow behavior of materials during metal cutting [16], which in turn affects chip flow patterns [17]. Davis et al. [18] employed in situ imaging technology to observe plastic flow changes during the cutting of tantalum-a material with high strain-hardening ability and low thermal conductivity-when its surface was coated with ink. They found that surface-active media could disrupt the sinuous flow on the workpiece surface and replace it with a favorable segmented flow pattern. Sugihara et al. [19] used high-speed in situ imaging to observe and analyze the ductile-to-brittle transition process of materials during cutting induced by the Rehbinder effect of surface-active media. Through molecular modeling and simulation, they demonstrated that the Rehbinder effect arises from surface stress changes induced by active media adsorbed on the workpiece surface.

Since the discovery of the Rehbinder effect in surface-active media, researchers have consistently utilized it to reduce cutting forces and plastic deformation of workpiece materials. Experimental studies have shown that coating specific surface-active media on metal surfaces results in significantly reduced cutting forces, improved machined surface quality, and decreased tool wear. Chaudhari et al. [20,21] applied ink as an active medium to coat hardened copper surfaces for turning, achieving a cutting force reduction of approximately 50% compared with uncoated conditions, and analyzed the underlying mechanism based on dislocation density theory. Zhang et al. [22,23] investigated the influence of the Rehbinder effect on the surface integrity of machined workpieces, proving that the Rehbinder effect can reduce the microhardness of the machined surface.

Researchers have conducted in-depth studies on the types of active media and found that the Rehbinder effect exists when various common media are coated on metal surfaces for cutting [24,25]. Udupa et al. [26,27] categorized surface-active media into two types: physical adsorption type (e.g., glue, ink) and chemical affinity type (e.g., ethanol, isopropanol), confirming their universality in the cutting of various metal materials. It has been demonstrated that using marking ink, vinyl pyridine alkyl glue, and ethanol as surface-active media can all reduce cutting forces during the machining of copper, aluminum, iron, pure nickel, and 304 stainless steel [28].

However, traditional surface-active media typically have a thermal conductivity below 0.1 W/m·K, which cannot address the core heat dissipation problem in Inconel 718 cutting, limiting their application in high-temperature alloy machining [29]. To overcome this critical limitation of poor thermal conductivity in traditional surface-active media, Yin et al. [30,31] proposed the concept of Active Thermal Conductive Medium coating (ATCM). By using high-thermal-conductivity active media, they achieved the synergistic coupling of the Rehbinder mechanochemical effect and solid-phase enhanced heat transfer effect. Through comparative experiments between ATCM with different thermal conductivities and dry cutting, liquid graphene was identified as the optimal ATCM for reducing both cutting temperature and cutting force in Inconel 718 machining.

Nevertheless, existing studies have only established performance comparisons between ATCM and dry cutting conditions, and have not systematically conducted horizontal comparisons between ATCM and widely used cooling and lubrication technologies. This has made it impossible to clarify the performance positioning and technical advantages of ATCM within the existing machining technology system.

Therefore, this study takes the optimized liquid graphene ATCM as the research object and conducts multi-condition comparative experiments including dry cutting, flood cutting, MQL, CMQL, NMQL, and ATCM-assisted cutting. Core performance indicators such as cutting force, cutting temperature, cutting power, and tool wear are measured, combined with microscopic characterization techniques including Scanning Electron Microscopy (SEM) and Energy Dispersive Spectroscopy (EDS), to compare the comprehensive machining performance of different strategies. On this basis, the essential differences between ATCM and conventional cooling/lubrication technologies in terms of cutting zone heat transfer modes, interfacial friction regulation mechanisms, and tool wear evolution laws are systematically revealed. This work provides a scientific basis for the selection and optimization of high-efficiency green machining technologies for nickel-based superalloys.

## 2. Materials and Methods

### 2.1. Materials and Pretreatment

The Inconel 718 workpieces used in the experiments were radial disks with a diameter of 130 mm and a thickness of 2 mm. The 130 mm outer diameter provides a complete continuous cutting stroke, which avoids transient signal disturbances generated during tool entry and retraction, and thus improves the reliability of force and temperature data collected under stable cutting states. First, the workpiece surfaces to be machined were polished to reduce the initial surface roughness, thereby minimizing the thermal contact resistance between the workpiece and various media. Subsequently, the workpieces were ultrasonically cleaned with anhydrous ethanol at a frequency of 40 kHz for 15 min, then dried and sealed inside a desiccator (Tenghui, Nanjing, China).

A series of comparative experiments were conducted to investigate the effects of different machining strategies (dry cutting, flood cutting, MQL cutting, CMQL cutting, NMQL cutting, and ATCM-assisted cutting) on the sustainable machining of Inconel 718 and elucidate the underlying mechanisms, with a focus on cutting force, cutting temperature, cutting power, surface roughness, and tool wear. Mineral oil and cottonseed oil were used as cutting fluids for flood cooling and MQL, respectively [32]. For CMQL, compressed air at a temperature of −20 °C was employed as the cooling medium. For NMQL, Al_2_O_3_ nanoparticles at a concentration of 1.5 wt% were mixed with cottonseed oil and 0.3 wt% sodium dodecyl sulfate (SDS) as a dispersant, followed by ultrasonic vibration for 20 min using a numerical control ultrasonic oscillator (Jiecheng, Shanghai, China) to prepare the nanofluid cutting fluid. For ATCM, liquid graphene was uniformly blade-coated on the workpiece surface at room temperature, and the samples were naturally dried in a clean Petri dish for 2 h before sealing. Graphene possesses an ultrahigh thermal conductivity of 5300 W/(m·K), enabling it to exert comprehensive surface effects including the Rehbinder effect and enhanced heat transfer.

### 2.2. Experiment Methods

**Figure 1:**
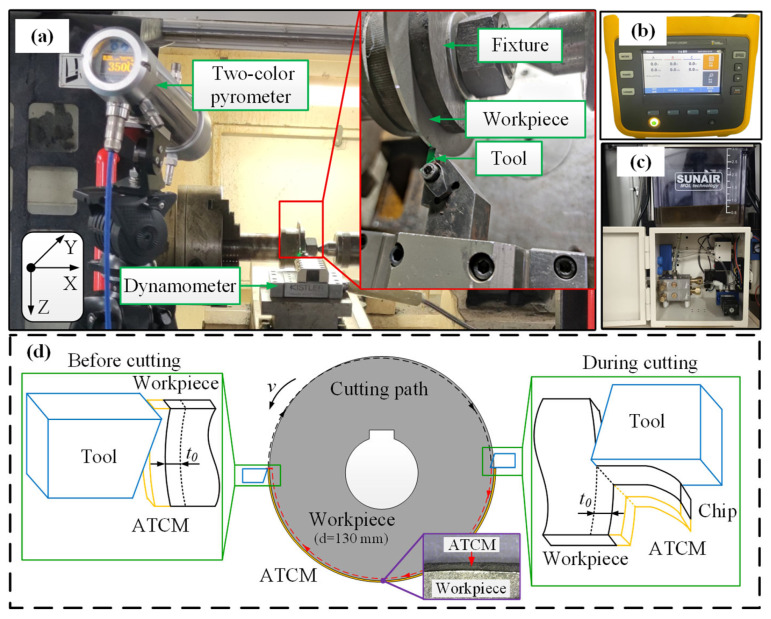
Experimental materials and process principle: (**a**) Experiment setup. (**b**) Three-phase power meter. (**c**) MQL setup. (**d**) Schematic diagram of cutting process.

The experimental setup was based on a CNC lathe, as shown in Figure 1a. Uncoated cemented carbide cutting inserts (K313, NG3189R, Kennametal, Pittsburgh, PA, USA) were used for machining, with cutting parameters set as follows: cutting speed *v* = 30 m/min, undeformed chip thickness *t*_0_ = 0.1 mm, and cutting width *a*_w_ = 2 mm. The average grain size of the WC hard phase is approximately 0.8 μm, and the mass fraction of the Co binder phase is 6%. A dynamometer (9257B, Kistler, Winterthur, Switzerland) mounted beneath the tool holder was used for continuous real-time measurement of cutting force variations throughout the process. A two-color pyrometer (STRON-GR-3514, Fluke, Everett, WA, USA) was employed to online measure the continuous changes in cutting temperature on the chip side surface. The two-color pyrometer has a measurement accuracy of ±0.1% tm °C (where tm is the upper limit of the temperature measurement range), a resolution of 0.1 °C, and a temperature measurement range of 350 °C to 1400 °C. A three-phase power meter (Fluke 1734, Fluke, Everett, WA, USA) was used to measure the power consumption during the entire cutting process, as shown in Figure 1b. Three independent repeated experiments were strictly conducted under completely consistent test parameters and environmental conditions for all working conditions.

For MQL conditions, a Sanai MQL system (Figure 1c) was used for lubrication, with jet parameters set to a flow rate of 50 mL/h and an air pressure of 0.5 MPa [9]. A vortex tube was installed on the MQL nozzle to realize CMQL machining. For workpieces coated with ATCM, the cutting path for orthogonal cutting of the radial disk is illustrated in Figure 1d. Before cutting, the ATCM-coated workpiece was aligned with the tool tip. The tool engagement phase, indicated by the black dashed line, corresponds to cutting on the uncoated portion of the workpiece surface. As the workpiece continues to rotate, the tool cuts into the ATCM-coated region (indicated by the red dashed line) at a cutting depth of 0.1 mm. The tool was retracted immediately after completing one full revolution of cutting.

After the cutting experiments, the cutting inserts were ultrasonically cleaned in anhydrous ethanol for 20 min, and the dried samples were stored for subsequent surface morphology observation and performance analysis. A field emission scanning electron microscope (FE-SEM, JSM-7610F, JEOL, Akishima, Japan) was used to characterize tool wear morphologies and perform energy-dispersive X-ray spectroscopy (EDS) elemental analysis of the tool surfaces.

## 3. Results

### 3.1. Cutting Force

**Figure 2:**
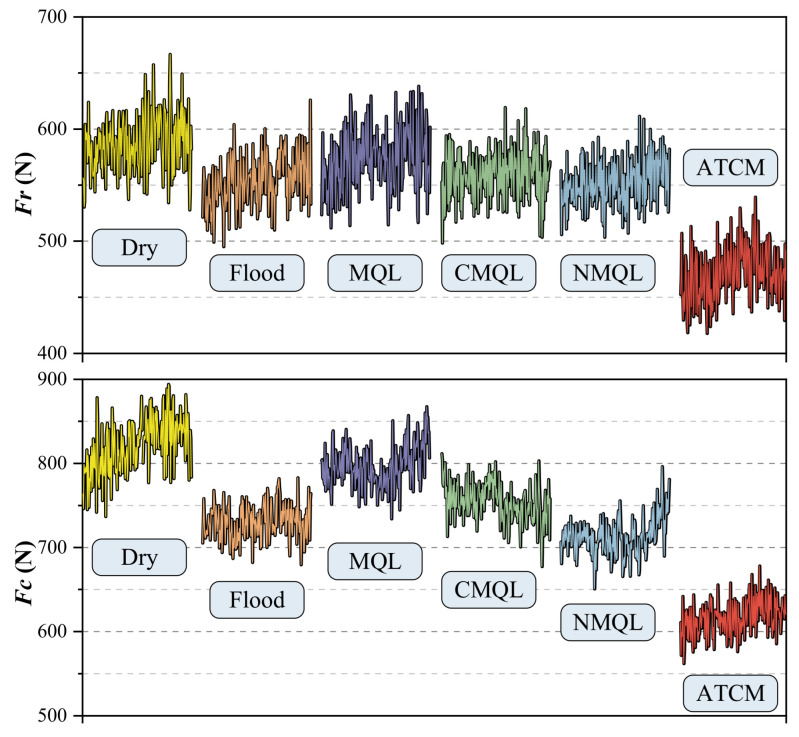
Raw cutting force data under different operating conditions.

**Figure 3:**
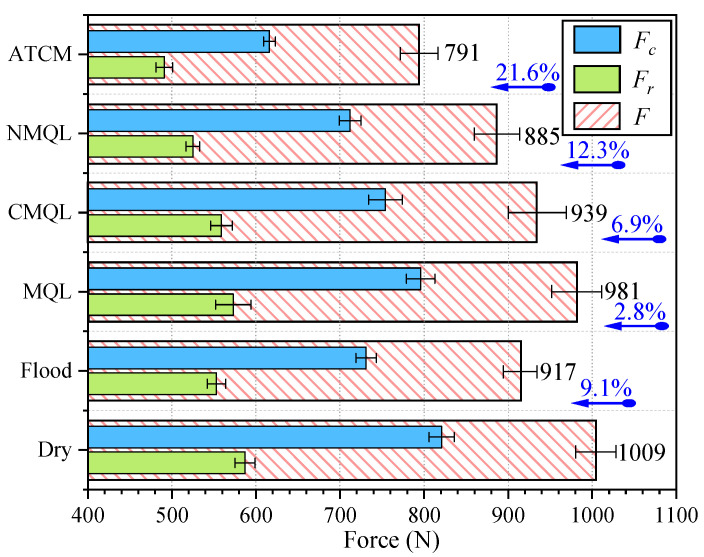
Tangential cutting, radial thrust, and resultant forces under different operating conditions.

The raw data of the tangential cutting force (Fc) and radial thrust force (Fr) recorded during the orthogonal cutting experiments are presented in Figure 2. All force measurements were taken during the steady-state cutting phase to eliminate transient effects during tool engagement. The resultant cutting forces, calculated from the measured tangential and radial force components, are summarized in Figure 3.

Dry cutting yielded the highest resultant force of 1009 N, imposing the most severe mechanical load on the tool-workpiece system. Flood cooling reduced the resultant force to 917 N, a 9.1% decrease compared to dry cutting. Conventional MQL showed limited improvement, with a resultant force of 981 N, only 2.8% lower than dry cutting. Among the conventional cooling/lubrication strategies, CMQL and NMQL achieved better force reduction performance. CMQL resulted in a resultant force of 939 N, corresponding to a 6.9% reduction relative to dry cutting, outperforming conventional MQL. NMQL further improved the force reduction effect, yielding a resultant force of 885 N (12.3% reduction vs. dry cutting).

Remarkably, ATCM-assisted cutting exhibited the most significant force reduction among all conditions, with a resultant force of 791 N. This represents a 21.6% decrease compared to dry cutting, and a 10.6% decrease compared to NMQL, the top-performing conventional strategy. The magnitude of force reduction achieved by ATCM exceeds that of other traditional cooling or lubrication methods.

### 3.2. Cutting Temperature

Cutting temperature is a critical parameter governing the machinability of Inconel 718, as it directly dictates tool wear evolution, built-up edge formation, and the surface integrity of machined components [33]. During orthogonal cutting, approximately 98% of the total mechanical energy input is converted into thermal energy, primarily generated by severe plastic deformation in the Primary Shear Zone (PSZ) and frictional interaction at the tool-chip interface in the Secondary Shear Zone (SSZ) [34]. Excessive localized temperatures can induce detrimental surface defects including high tensile residual stresses and brittle white layers, which significantly compromise the fatigue life and reliability of aero-engine hot-end components. Therefore, quantitative characterization of cutting temperatures under different cutting conditions is essential for evaluating their thermal load mitigation capabilities.

**Figure 4:**
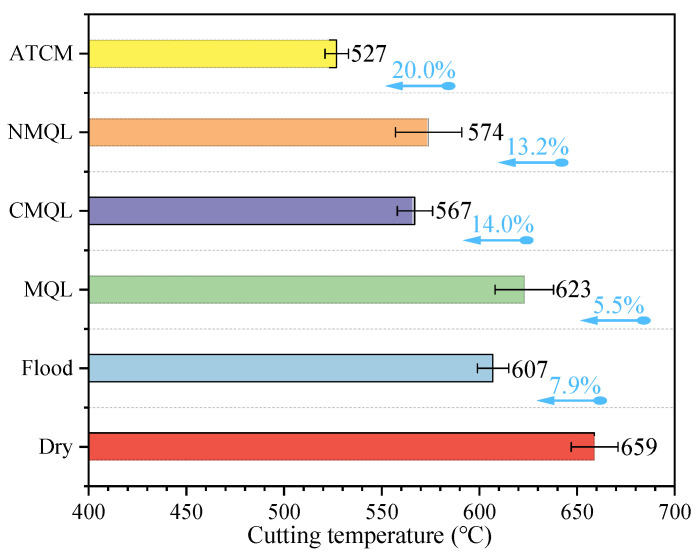
Cutting temperature under different operating conditions.

The measured steady-state cutting temperatures under various machining strategies are presented in Figure 4. Temperature measurements were performed on the free side surface of the chip using a calibrated two-color pyrometer, with each experimental condition repeated three times to ensure statistical reliability. The reported values represent the average peak temperatures recorded during the stable cutting phase, with error bars indicating the standard deviation of the measurements.

Dry cutting yielded the highest cutting temperature of 659 °C, as the only heat dissipation pathway was natural convection through air, which has an extremely low thermal conductivity of 0.025 W/m·K. Flood cooling, which delivers a high volume of cutting fluid for forced convection, reduced the cutting temperature to 607 °C, corresponding to a 7.9% decrease compared to dry cutting. Conventional MQL showed limited cooling effectiveness due to the minimal volume of atomized lubricant delivered to the cutting zone, resulting in a cutting temperature of 623 °C, only 5.5% lower than dry cutting.

Among the conventional enhanced cooling strategies, CMQL and NMQL achieved more significant thermal load reductions. CMQL, which integrates MQL with −20 °C cryogenic compressed air, resulted in a cutting temperature of 567 °C, representing a 14.0% reduction relative to dry cutting. The nanoparticles in NMQL can achieve enhanced convective heat transfer, resulting in a cutting temperature of 574 °C (12.9% reduction vs. dry cutting). ATCM-assisted cutting achieved the lowest cutting temperature among all cutting conditions at 527 °C. This represents a 20.0% reduction compared to dry cutting, and a 7.1% reduction compared to CMQL-the best-performing conventional cooling strategy.

### 3.3. Cutting Power

The cutting power was measured using a two-cycle measurement method, as detailed in Equation (1). This method requires two separate power measurements under identical cutting parameters: one during air cutting (no material removal) to obtain the idle power P_air, and another during actual cutting to obtain the total power consumption P_total. The net cutting power P_cut, representing the actual power consumed by the material removal process, is calculated as the difference between these two values.

Specific cutting energy (SCE), defined as the energy required to remove a unit volume of material, was further derived from the net cutting power using Equation (2) in units of J/mm^3^. SCE encompasses three primary energy components: plastic deformation in the primary deformation zone, interfacial friction between the tool and workpiece, and the formation of new machined surfaces [35]. As a fundamental quantitative indicator of machinability, SCE characterizes the magnitude of energy transfer during cutting and serves as a key metric for evaluating machining efficiency. Lower SCE values correspond to higher material removal efficiency, reduced energy consumption, and improved environmental sustainability [36].

A single-factor experimental design was employed in this study, where only the cooling/lubrication conditions were varied while the material removal rate (MRR) was maintained constant across all cutting conditions. Therefore, direct comparison of cutting power under different conditions provides a valid and straightforward assessment of relative machining efficiency.

(1) $$P_{cut}=P_{total}-P_{air},$$

(2) $$SCE=\frac{P_{cut}}{MRR}=\frac{P_{cut}}{f\times {\;v}_{c}\times t_{0}}$$

**Figure 5:**
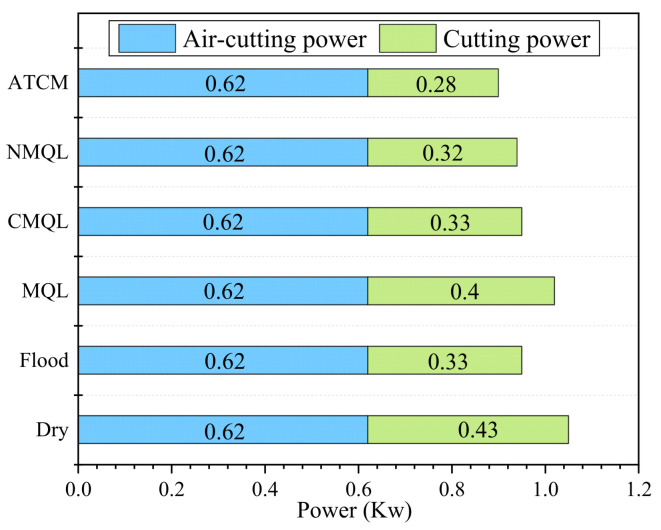
Cutting power under different operating conditions.

The cutting power obtained under different cutting conditions is shown in Figure 5, and there is a significant change in cutting power under different processing conditions. Dry cutting exhibited the highest cutting power consumption, corresponding to the maximum energy input required for material removal. ATCM-assisted cutting achieved the lowest cutting power, representing a 34.9% reduction compared to dry cutting. NMQL ranked second, with a 25.6% reduction in cutting power relative to dry cutting. CMQL and flood cooling yielded nearly identical cutting power values, both showing approximately 22% reduction compared to dry cutting. Conventional MQL showed the least improvement among all cutting strategies, with only a 7.0% reduction in cutting power compared to dry cutting. These results demonstrate that ATCM significantly outperforms all conventional cooling and lubrication technologies in terms of energy efficiency, highlighting its substantial potential for sustainable machining of Inconel 718.

### 3.4. Tool Wear

**Figure 6:**
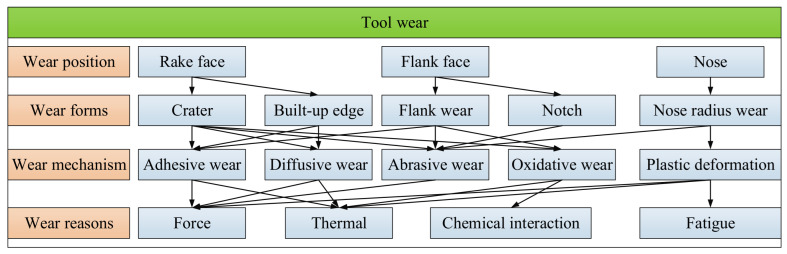
Tool wear mechanism.

Inconel 718 is a typical difficult-to-machine material, and the high temperature and pressure in the cutting zone during machining will cause severe wear on the rake and flank faces of the cutting tool [37]. Tool wear not only shortens the service life of the cutting tool but also directly affects the dimensional accuracy of the workpiece and the surface quality of the machined surface [38]. The wear of cutting tools is a complex coupling process of thermo-mechanical loads and chemical reactions, involving multiple wear mechanisms that interact and promote each other, as shown in Figure 6.

#### 3.4.1. Rake Face Wear Morphology

Given the relatively short cutting duration in the orthogonal cutting experiments, flank wear was not significant. Therefore, tool wear was primarily evaluated based on the morphology of the rake face, which serves as the primary contact interface between the cutting tool and the chip.

**Figure 7:**
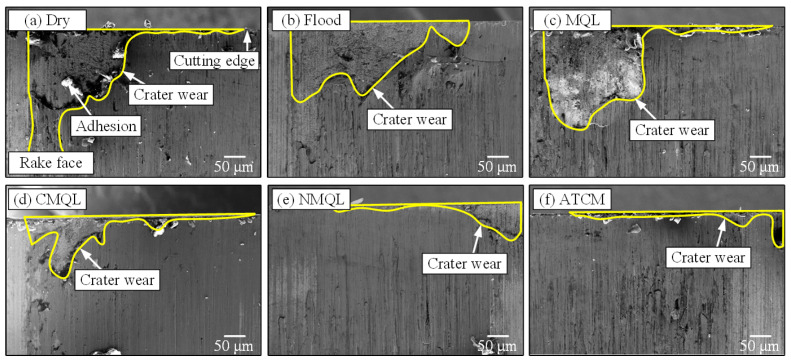
Adhesive wear on the rake face of the cutting tool.

Figure 7 presents the SEM micrographs of rake face wear under different machining conditions. Irregular wear regions were observed near the tool tip in all cases, with clear morphological distinctions between the worn and unworn areas. Adhesive wear was identified as the dominant wear mechanism on the tool-chip contact interface across all conditions. Adhesive wear arises from severe extrusion and frictional interaction between the tool, chip, and workpiece during cutting, with its severity directly correlated to the magnitude of thermo-mechanical loads.

The wear morphologies of the tool rake faces under different machining conditions are presented in Figure 7. Irregular wear regions were observed near the tool tip, with distinct morphological differences between the worn and unworn areas. Adhesive wear was identified as the dominant wear mechanism on the tool-chip contact interface across all cutting conditions. Adhesive wear arises from severe extrusion and frictional interaction between the tool, chip, and workpiece during cutting, and its severity is directly proportional to the magnitude of cutting forces and temperatures [39]. When the thickness of the adhered workpiece material reaches a critical value, it cracks and undergoes delamination under the continuous friction, extrusion, shear, and impact forces from subsequent cutting passes. During this delamination process, portions of the tool substrate are torn away by the adhered material, leading to progressive tool material loss. The exposed fresh tool surface is then immediately re-adhered by workpiece material, creating a cyclic adhesion-delamination process that results in severe adhesive wear during Inconel 718 machining.

Dry cutting exhibited the largest adhesive wear area, accompanied by extensive material adhesion. As thermo-mechanical loads decreased with improved cooling and lubrication strategies, crater wear on the rake face was mitigated to varying degrees. MQL and flood cooling showed the least improvement in adhesive wear. CMQL, benefiting from the cryogenic environment, significantly reduced the wear area. Notably, NMQL and ATCM achieved the lowest levels of adhesive wear, attributed to their substantial reductions in both cutting force and temperature.

**Figure 8:**
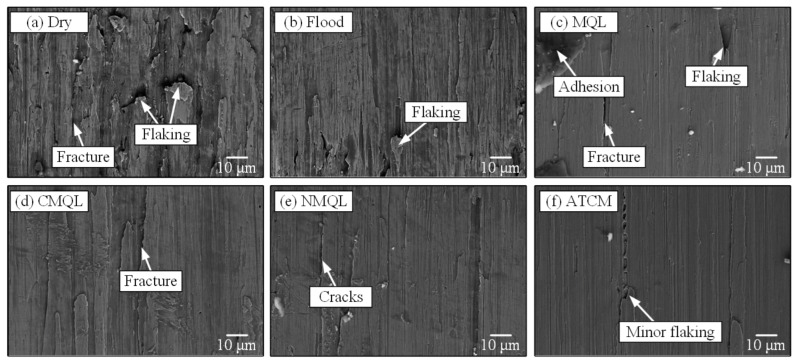
Defects on the front cutting surface of the tool.

Further high-magnification observations, as shown in Figure 8, revealed severe abrasive wear during the cutting process, with numerous parallel ridges and grooves formed by abrasive wear on the tool rake faces. The abrasive particles originated primarily from two sources: hard carbide precipitates (e.g., TiC, NbC) inherent in the Inconel 718 matrix and WC particles detached from the cemented carbide tool substrate. Under the high-temperature and high-pressure conditions in the cutting zone, severe friction and extrusion between the chip and tool exacerbated tool wear, causing extensive abrasive interaction between these hard carbide particles and the tool rake face and cutting edge. As these hard abrasive particles slide across the tool surface, they induce plastic deformation or fracture of the tool material, resulting in the formation of characteristic scratches and grooves.

As shown in Figure 8a, catastrophic fracture and severe lamellar flaking were observed on the rake face under dry cutting conditions, representing one of the most severe forms of tool failure. The formation of surface craters and lamellar wear was primarily attributed to the adiabatic impact effect at the tool-chip interface. As chips flow along the tool rake face, they impact the tool surface with an extremely short interaction time. The heat generated at the tool-chip interface cannot dissipate rapidly, resulting in the formation of localized adiabatic impact zones on the tool surface. The adiabatic impact causes a rapid temperature rise on the tool surface, which significantly increases the plastic deformability of the tool material. The chip impact induces micro-deformation of the tool surface, forming craters and lip-shaped protrusions. Under continuous chip impact, cracks initiate on the worn tool surface with craters and lips. These cracks propagate, coalesce, and fracture through the tool surface and subsurface, ultimately leading to lamellar material removal. Delamination of adhered workpiece material from the tool surface further reduces the surface strength of the tool, contributing to additional lamellar flaking.

Similar catastrophic fracture phenomena were also observed under MQL and CMQL conditions due to insufficient lubrication. Inadequate cooling effectiveness under flood and MQL conditions also resulted in lamellar flaking on the tool rake face. In contrast, under NMQL and ATCM conditions, the significantly reduced thermo-mechanical loads resulted in only minor defects on the tool rake face, including microcracks and slight flaking.

#### 3.4.2. EDS Elemental Analysis

**Figure 9:**
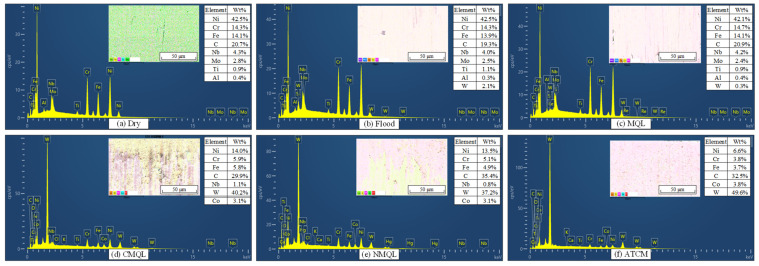
EDS analysis of the front cutting surface of the tool.

Comparative energy-dispersive X-ray spectroscopy (EDS) analysis of the tool rake faces under the six cutting conditions, as presented in Figure 9, revealed high concentrations of workpiece elements (Ni, Fe, Cr) and tool substrate element (W) in the wear regions. This indicates the concurrent occurrence of significant oxidative wear and diffusion wear during cutting, which degrade the tool performance. Under dry, flood, and MQL conditions, the total weight percentage of Ni, Fe, and Cr detected on the worn tool surfaces reached approximately 70%, confirming extensive interdiffusion between the tool and workpiece during the machining of nickel-based superalloys. The high cutting temperatures generated during Inconel 718 machining, combined with the high-pressure conditions at the contact interfaces, promote interdiffusion of Fe, Ni, and Cr from the workpiece and Co from the tool substrate across the grain boundaries into each other’s surface layers. According to Fick’s law, the extent of diffusion is governed by the diffusion coefficient, which is primarily dependent on the cutting temperature [40]. Therefore, different machining conditions have a significant impact on the degree of diffusion, leading to varying levels of workpiece element penetration into the tool surface.

Cobalt (Co), the binder phase in cemented carbide tools, and nickel (Ni) and iron (Fe) from the Inconel 718 workpiece are elements of the same group in the periodic table, possessing similar crystal structures and exhibiting certain chemical affinity. During cutting, concentration gradients exist between Co in the tool and its homologous elements in the workpiece, which drive atomic diffusion. Driven by chemical potential differences, workpiece elements such as Ni, Fe, and Cr diffuse into the tool substrate. This atomic-scale migration directly reduces the hardness and strength of the tool matrix. Under the continuous action of shear forces and thermo-mechanical loads, cracks initiate on the tool rake face, and their propagation ultimately leads to large-scale lamellar flaking. Additionally, the cyclic adhesion and delamination of Inconel 718 on the tool surface further weaken the strength of the tool surface layer. Diffusion wear requires two prerequisite conditions: intimate contact at the tool-chip and tool-workpiece interfaces, and elevated cutting temperatures, with the latter being the dominant factor influencing the severity of diffusion wear. As cutting conditions improve and cutting temperatures decrease, the diffusion phenomenon is significantly mitigated. Under CMQL, NMQL, and ATCM conditions, the concentrations of Ni, Fe, and Cr on the tool surface decreased, while the weight percentages of W detected were 40.2%, 37.2%, and 49.6%, respectively. This is attributed to both the reduced diffusion caused by lower cutting temperatures and the diminished material adhesion under these conditions, which collectively result in lower workpiece element content and higher W content on the tool rake face.

In summary, tool wear during Inconel 718 cutting is a complex multi-mechanism coupled evolution process dominated by adhesive wear and abrasive wear, accompanied by diffusion wear at elevated temperatures. All wear mechanisms interact and reinforce each other, with the thermo-mechanical loads in the cutting zone serving as the core driving force for the initiation and progression of these mechanisms. ATCM achieves the most effective control of tool wear among all cutting conditions by simultaneously reducing cutting forces and temperatures through the synergistic effect of the Rehbinder effect and enhanced heat transfer, which fundamentally weakens the driving forces for all wear mechanisms.

## 4. Discussion

### 4.1. Lubrication Mechanism and Rehbinder Effect

**Figure 10:**
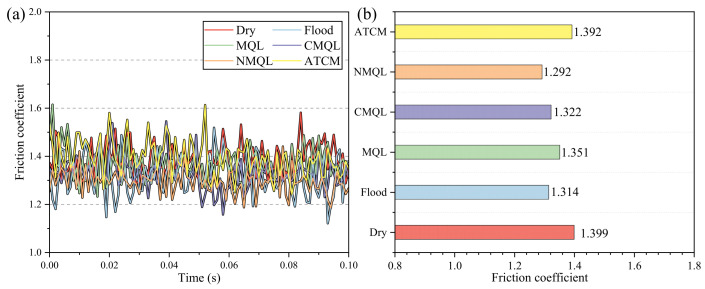
Coefficient of friction under different cutting conditions, (**a**) curve; (**b**) average.

The frictional behavior at the tool-chip and tool-workpiece interfaces during cutting directly governs cutting forces, energy consumption, and tool wear behavior, making it the core factor influencing the machinability of Inconel 718. Therefore, to evaluate the tribological performance of the cutting tool under different conditions, the coefficient of friction (μ) was calculated using the ratio of the main cutting force to the radial thrust force, as shown in Figure 10.

Under dry cutting conditions, no lubricating medium was present at the cutting interface, resulting in direct metal-to-metal contact between the tool and workpiece and a state of dry friction. The interface friction coefficient was the highest among all conditions at 1.399. The severe interfacial friction led to the maximum resultant cutting force and extensive adhesive wear on the tool rake face.

Flood cutting provided an abundant supply of cutting fluid, which could penetrate into the micro-gaps at the tool-chip interface and form a continuous lubricating oil film. This transformed the interfacial dry friction into fluid lubrication friction, thereby significantly reducing the interface friction coefficient to 1.314. Consequently, the resultant cutting force under this condition was 9.1% lower than that under dry cutting. However, under the high-temperature and high-pressure environment of Inconel 718 cutting, the lubricating oil film was highly susceptible to thermal degradation and rupture, making it difficult to achieve full-film lubrication and limiting the friction reduction effect.

MQL conditions relied on a minimal amount of atomized cutting fluid to form a lubricating film at the interface [41]. The reduced cutting fluid volume led to decreased coverage area and load-bearing capacity of the oil film, resulting in frequent local dry friction at the interface. Therefore, its lubrication effect was far inferior to that of flood cutting, with a friction coefficient of 1.351. This also explains the negligible improvement in tool adhesive wear observed under MQL conditions.

CMQL exhibited a moderate improvement in lubrication effect compared to MQL, primarily due to the cryogenic cold air reducing the cutting zone temperature, inhibiting thermal degradation of the lubricating oil film, and enhancing its stability and continuity. Under the cryogenic effect, the friction coefficient of CMQL was 1.322, and the resultant cutting force was 6.9% lower than that under dry cutting. Nevertheless, it was still limited by the film-forming ability of atomized droplets, resulting in a weaker friction reduction effect than flood cutting.

**Figure 11:**
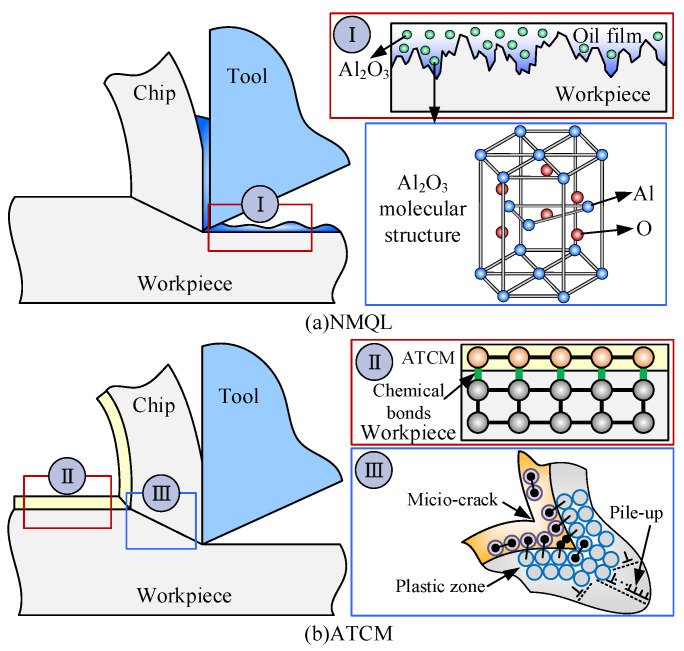
Ball-bearing effect of nanoparticles in NMQL and Rehbinder effect of ATCM.

Benefiting from the multi-scale lubrication effect of Al_2_O_3_ nanoparticles, NMQL achieved the lowest friction coefficient of 1.292 among all conventional lubrication strategies. This was mainly attributed to the hexagonal close-packed molecular structure of spherical Al_2_O_3_ nanoparticles, which possess excellent chemical stability, high strength, and a high melting point. These nanoparticles acted as “micro ball bearings” at the tool-chip and tool-workpiece interfaces, converting interfacial sliding friction into rolling friction and directly reducing the friction coefficient. Additionally, the nanoparticles could fill micro-pits at the interface, repair defects in the lubricating oil film, and improve its load-bearing capacity and continuity, as shown in Figure 11a. The physical protective film formed by nanoparticles deposited on the tool and workpiece surfaces prevented direct metal-to-metal contact at the interface, reducing the occurrence of tool adhesive wear.

A fundamental limitation of conventional lubrication technologies is their inability to form stable lubricating films at high-temperature and high-pressure interfaces, which ultimately leads to tool adhesive wear. In contrast, the cutting force reduction effect of ATCM originates from the Rehbinder effect of the surface-active medium rather than interfacial lubrication and friction reduction, representing an essentially different mechanism from traditional lubrication methods. ATCM is pre-coated on the workpiece surface to be machined and is removed synchronously with the workpiece material during cutting. It cannot enter the tool-chip and tool-workpiece contact interfaces to form a lubricating oil film, thus having no direct effect on the lubrication performance of the cutting interface. As shown in Figure 10, the friction coefficient under ATCM conditions was 1.392, which was comparable to that under dry cutting.

Unlike traditional lubrication mechanisms, the Rehbinder effect improves machinability through mechanochemical modification of the workpiece surface layer. Traditional lubrication technologies can only reduce the frictional resistance at the tool-chip and tool-workpiece interfaces but have no effect on the plastic deformation resistance of materials in the Primary Shear Zone (PSZ). In contrast, the Rehbinder effect directly alters the physical and mechanical properties of the workpiece surface layer to be machined, reducing the energy required for material removal from the source of cutting deformation, as illustrated in Figure 11b. Its specific mechanism operates at two levels: First, the polar groups of the surface-active molecules in ATCM form chemical bonds with the stable oxide film on the workpiece surface, promoting chemisorption while reducing the material’s surface free energy. This enhances surface dislocation mobility, facilitates slip deformation of workpiece atoms, and induces dislocation pile-up and strain localization within grains, ultimately leading to ductile-to-brittle transition of the material and significantly reducing the critical shear stress required for shear slip in the PSZ. Second, the Rehbinder effect modifies the plastic flow behavior of the material during cutting, suppresses sinuous flow, reduces the adhesion tendency of workpiece material at the tool-chip interface, and decreases the additional shear resistance at the interface. Consequently, the cutting force under ATCM conditions was significantly reduced by 21.6% compared to dry cutting, achieving the best performance among all tested conditions.

### 4.2. Enhanced Heat Transfer Mechanisms

**Figure 12:**
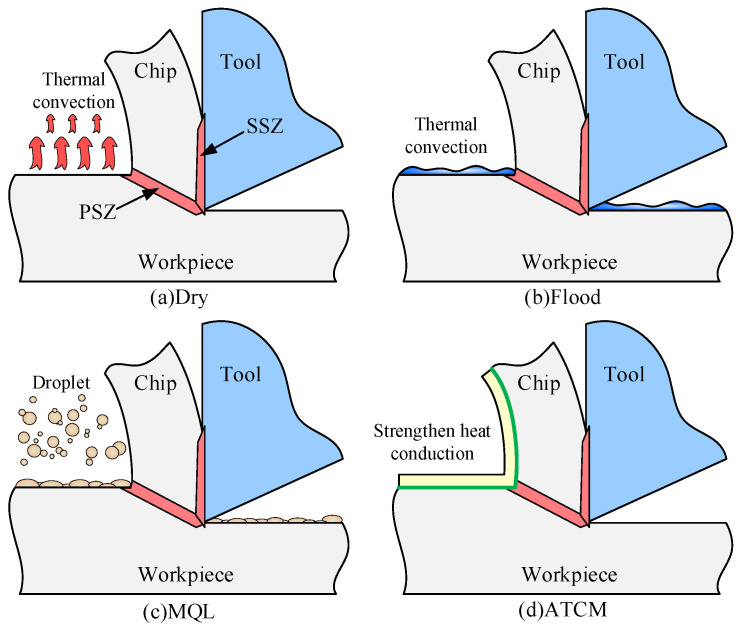
Heat dissipation mechanisms under different cutting conditions.

During the cutting of Inconel 718, approximately 80% of the cutting heat originates from shear slip plastic deformation of the material in the PSZ, about 18% comes from tool-chip interface friction in the SSZ, and less than 2% is attributed to elastic recovery friction in the tertiary deformation zone, which is negligible. The heat dissipation performance under different cutting conditions is essentially determined by their heat transfer modes and efficiencies, as illustrated in Figure 12.

Under dry cutting conditions, heat dissipation relies solely on natural convection of air, which has an extremely low thermal conductivity of only 0.025 W/m·K, providing almost no enhanced heat transfer effect. The cutting heat generated in the PSZ and SSZ cannot be rapidly conducted away through the workpiece matrix, and no additional heat dissipation channels are available, resulting in complete heat accumulation in the cutting zone. Consequently, the cutting temperature under this condition reaches 659 °C, the highest among all tested conditions. The extreme localized high temperature further reduces the thermal conductivity of the Inconel 718 workpiece, forming a positive feedback loop of “heat accumulation—thermal conductivity degradation—further temperature rise”. This is the core reason why both cutting forces and tool wear are the most severe under dry cutting.

Flood cutting achieves heat dissipation through forced convection of a large volume of cutting fluid, as shown in Figure 12b. However, its heat transfer efficiency is severely limited by the Leidenfrost effect. When the cutting fluid contacts the high-temperature tool-chip interface, it instantaneously vaporizes to form a continuous vapor film, which isolates the liquid-phase cutting fluid from direct contact with the high-temperature interface, leading to a sharp decay in convective heat transfer efficiency. Therefore, despite the abundant supply of cooling medium, the cutting temperature under flood cutting is only 607 °C, representing a mere 7.9% reduction compared to dry cutting.

MQL conditions utilize jet impingement heat transfer of atomized cutting fluid droplets, as shown in Figure 12c. The limited number of atomized droplets cannot form a continuous liquid film at the high-temperature interface, resulting in significantly weaker convective heat transfer capability than flood cutting. The final cutting temperature is 623 °C, only 5.5% lower than that under dry cutting. This result reveals the core shortcoming of MQL in Inconel 718 machining: relying solely on a trace amount of atomized coolant cannot meet the high heat flux dissipation requirements during the cutting of this material.

CMQL introduces −20 °C cryogenic cold air on the basis of MQL, and its enhanced heat transfer effect is manifested in two aspects: First, according to Newton’s law of cooling, convective heat transfer capability is positively correlated with the temperature difference between the heat transfer medium and the cutting zone. The cryogenic cold air significantly increases this temperature difference, directly enhancing the forced convective heat transfer efficiency. Second, the low-temperature environment suppresses the vaporization of cutting fluid droplets, alleviates the Leidenfrost effect at the high-temperature interface, and improves the effective contact rate between the liquid-phase coolant and the heat source. Under the synergistic effect of these two mechanisms, the cutting temperature under CMQL conditions drops to 567 °C, a 14% reduction compared to dry cutting, with heat dissipation performance significantly superior to both MQL and flood cutting.

NMQL incorporates Al_2_O_3_ nanoparticles into the MQL cutting fluid and achieves enhanced heat transfer through two mechanisms: On one hand, the high thermal conductivity of nanoparticles significantly improves the effective thermal conductivity of the base fluid, while the Brownian motion of nanoparticles enhances energy exchange at the liquid–solid interface. On the other hand, nanoparticles deposited at the interface can increase boundary layer disturbance and reduce the thermal resistance of the laminar sublayer. Consequently, the cutting temperature under NMQL conditions is 574 °C, a 12.9% reduction compared to dry cutting, with heat dissipation performance better than MQL but slightly inferior to CMQL. The core reason is that NMQL is still limited by the total heat capacity of the atomized droplets and cannot completely eliminate the barrier effect of the vapor film at the high-temperature interface, making it difficult to further improve heat dissipation efficiency.

Unlike convective heat transfer, the use of liquid graphene (with an ultrahigh thermal conductivity of 5300 W/m·K) as ATCM forms a “workpiece-chip-ATCM film” solid-phase heat conduction channel on the workpiece surface, as shown in Figure 12d. This breaks through the technical bottleneck of traditional convective heat transfer limited by the Leidenfrost effect. From the perspective of heat conservation and heat distribution mechanisms, the heat dissipation paths of traditional cooling methods are limited to forced convection between the coolant and the outer surfaces of the tool and chip, and cannot directly act on the heat sources inside the shear zones. In contrast, ATCM is pre-coated on the workpiece surface to be machined and intimately adheres to both the workpiece matrix and the newly formed chip surfaces, providing additional rapid heat dissipation channels for the cutting heat generated in the PSZ and SSZ: A portion of the heat transferred to the workpiece matrix is rapidly conducted laterally through the ATCM film, avoiding heat accumulation in the workpiece surface layer. Meanwhile, the heat inside the chip can be rapidly conducted away along the ATCM film on the chip surface, significantly increasing the proportion of heat carried away by the chips. This solid-phase heat transfer mode, which directly acts on the heat sources, avoids the thermal resistance caused by the vapor film in convective heat transfer, ultimately achieving the lowest cutting temperature of 527 °C, a 20% reduction compared to dry cutting.

The enhanced heat transfer effect of ATCM forms a positive synergistic coupling with the Rehbinder effect induced by the surface-active medium. The Rehbinder effect reduces the plastic deformation work and friction work during cutting by promoting the ductile-to-brittle transition of the workpiece surface layer, thereby minimizing the generation of cutting heat at the source; while the high-thermal-conductivity film enables efficient dissipation of the generated cutting heat, further lowering the temperature in the cutting zone. This synergistic effect of reduced heat generation and enhanced heat dissipation enables simultaneous optimal control of both cutting temperature and cutting force.

## 5. Conclusions

To address the core challenges of concentrated thermo-mechanical loads, severe tool wear, and poor machining quality in the cutting of Inconel 718, a green machining method based on ATCM is proposed in this study. The cutting performances under six machining conditions are systematically compared, and the coupled mechanochemical and heat transfer regulation mechanism of ATCM is revealed. The experimental results agree well with the expected outcomes, the main conclusions are drawn as follows:

(1) ATCM achieves optimal control of thermo-mechanical loads in the cutting zone through the synergistic coupling of the Rehbinder effect and enhanced heat transfer effect. Compared with dry cutting, ATCM reduces the cutting force by 21.6%, cutting temperature by 20%, and cutting power by 34.9%.
(2) The interfacial friction regulation mechanisms of different machining strategies are elucidated. Conventional lubrication strategies reduce interfacial friction by forming lubricating oil films, among which NMQL achieves the lowest friction coefficient (1.292) due to the “micro ball-bearing” effect of nanoparticles. In contrast, the cutting force reduction effect of ATCM originates entirely from the Rehbinder effect, which fundamentally reduces the energy required for material removal by inducing ductile-to-brittle transition of the workpiece material.
(3) The solid-phase enhanced heat transfer mechanism of ATCM is revealed. Liquid graphene ATCM constructs a “workpiece-chip-ATCM film” solid-phase heat conduction channel that directly acts on the cutting heat sources, avoiding the limitation of the Leidenfrost effect in convective heat transfer and realizing efficient dissipation of cutting heat.
(4) By reducing thermo-mechanical loads in the cutting zone, ATCM effectively inhibits the initiation and progression of adhesive wear, abrasive wear, and diffusion wear, prevents severe tool failure modes such as lamellar flaking. Currently, the ATCM process is suitable for small-batch, high-precision finishing applications, while its practical applicability for mass production remains to be further improved.

## Data Availability

The original contributions presented in this study are included in the article. Further inquiries can be directed to the corresponding author.

## Abbreviations

The following abbreviations are used in this manuscript:

ATCM	Active Thermal Conductive Media
MQL	Minimum Quantity Lubrication
CMQL	Cryogenic Minimum Quantity Lubrication
NMQL	Nanofluid Minimum Quantity Lubrication
SEM	Scanning Electron Microscopy
EDS	Energy Dispersive Spectroscopy
SCE	Specific cutting energy
MRR	Material removal rate
PSZ	Primary Shear Zone
SSZ	Secondary Shear Zone
